# Bioinorganic Synthesis of Sodium Polytungstate/Polyoxometalate in Microbial Kombucha Media for Precise Detection of Doxorubicin

**DOI:** 10.1155/2022/2265108

**Published:** 2022-08-08

**Authors:** Seyyed Mojtaba Mousavi, Seyyed Alireza Hashemi, Sonia Bahrani, Asma Sadrmousavi-Dizaj, Omid Arjmand, Navid Omidifar, Chin Wei Lai, Wei-Hung Chiang, Ahmad Gholami

**Affiliations:** ^1^Department of Chemical Engineering, National Taiwan University of Science and Technology, Taipei, Taiwan; ^2^Nanomaterials and Polymer Nanocomposites Laboratory, School of Engineering, University of British Columbia, Kelowna, Canada; ^3^Biotechnology Research Center, Shiraz University of Medical Science, Shiraz, Iran; ^4^Department of Physical Chemistry, University of Tabriz, Tabriz 5166616471, Iran; ^5^Department of Pathology, School of Medicine, Shiraz University of Medical Sciences, Shiraz, Iran; ^6^Nanotechnology and Catalysis Research Centre (NANOCAT), Level 3, Block A, Institute for Advanced Studies (IAS), University of Malaya (UM), Kuala Lumpur 50603, Malaysia; ^7^Pharmaceutical Sciences Research Center and Department of Pharmaceutical Biotechnology, School of Pharmacy, Shiraz University of Medical Science, Shiraz, Iran

## Abstract

In this study, we have developed a new platform of polyoxometalate as a biocompatible and electrosensitive polymeric biosensor for the accurate detection of doxorubicin. For this purpose, we used a green synthesis approach using tartaric acid, glutamic acid, and kombucha solvent. Thanks to its bioinorganic components, the biogenic approach can chemically modify and improve the performance of the biosensor, which was experimentally confirmed. Our results showed excellent sensitivity (175.72 *μ*A·*μ*M^−1^·cm^−2^), low detection limit (DL, 8.12 nM), and low quantification limit (QL, 0.056 *μ*M) when the newly developed biosensor was used. The results also show that the biosynthesized biosensor has improved performance in detecting DOX in the biological fluid with an accuracy of more than 99% depending on the components used, which underlines the high efficiency of the biosensor produced. Considering the body's physiological condition, the biosensor fabricated as a biocompatible component can show high efficiency. Therefore, its applicability for clinical use still needs to be studied in detail.

## 1. Introduction

Doxorubicin (DOX) is a chemotherapeutic agent used under the brand name Adriamycin to treat a variety of cancers in humans. It is used to treat different types of cancer, such as lung cancer, sarcoma, Hodgkin's/non-Hodgkin's lymphoma, breast cancer, and leukemia. DOX helps treat cancer through several mechanisms. Its main anticancer properties are the degradation of DNA by inhibiting topoisomerase II and the production of free radicals [1]. It has been shown that the use of this drug leads to common side effects such as bone marrow aplasia, alopecia, stomatitis, vomiting, gastrointestinal disorders, neurological disorders (hallucinations, dizziness, lightheadedness), and acute nausea due to toxicity in healthy tissues or resistance of tumor cells. Of all the side effects, cardiotoxicity is the most common complication limiting DOX. Controlling DOX concentrations in biological fluids (e.g., blood) during chemotherapy is critical to minimizing side effects. Identifying DNA damage and detecting DOX in biological fluids is applied by analytical methods such as photoelectrochemistry, mass spectrometry, capillary zone, capillary liquid chromatography, electrophoresis, and fluorescence. Although these analytical methods are accurate, they have disadvantages such as high cost and long testing times. As reported and confirmed in the literature, the electrochemical method has a high potential for DNA damage detection and is an attractive alternative approach for damage detection that has recently received more attention compared to other methods [2]. Polyoxometallates (POMs) are metal oxide aggregates with a great diversity in their structure and composition [3]. Their unique inherent properties make them promising candidates for medicine, magnetism, materials science, optics, biotechnology, and environmental sensing [4–6]. Many researchers have focused on studying polyoxometalates in medicinal chemistry [7, 8]. Many POMs, such as organotitanium-substituted heteropolytungstate, PM-26, PM-17, and PM-32, showed antitumor properties against various cancers in vitro or in vivo [9–11]. ATP generation prevented the formation of the 6-FMN (Mo_7_O_24_) complex in tumor cell mitochondria, resulting in antitumor activity. Prudent et al. found that P_2_Mo_18_O_62_ inhibits the protein kinase CK2 [12]. In addition, Müller et al. reported that K_6_H_2_ [TiW_11_CoO_40_] could be used as an inhibitor [13], as POMs can interact with proteins, although their anticancer mechanisms have not been extensively studied. Bioimprovement of polymeric structures such as POM can enhance their performance and biocompatibility.

Biosensors have attracted considerable attention due to their potential applications, including clinical diagnostics, forensic investigations, and environmental monitoring [14, 15]. Advancements in DNA biosensors can be achieved by developing surface DNA probe immobilization in conjunction with hybridization techniques between complementary DNA sequences [16, 17]. Many studies have been conducted with extensive reports on the characterization of DNA probe immobilization [18]. DNA biosensors have a very high sensitivity to direct detection of DNA structures. In addition, signal amplification strategies can increase the sensitivity of electrochemical biosensors [19, 20]. To monitor DOX that has entered clinical blood samples, Chekin et al. designed and fabricated a sensitive sensor with high efficiency based on nitrogen-doped reduced graphene oxide and chitosan as a biocompatible natural polymer [21, 22]. The research conducted by Alarfaj et al. showed that a NiO/MgO nanocomposite modified with a wire sensor has a high potential for determining DOX in human plasma, which was experimentally demonstrated [23]. Gardikis et al. presented a novel and effective drug carrier formed by using the bacterium *Lactobacillus helveticus* as a microbial biosensor to monitor the encapsulation efficiency of DOX [24]. The probiotic-based approach can perform well clinically as it is considered a suitable biosensor as it shows good symbiosis throughout the human body [25]. Behravan et al. used an electrochemical sensor based on a nanocomposite of modified glassy carbon electrodes (GCE), gold (Au) nanoparticles, reduced graphene oxide (rGO), and polypyrrole (PPy) for the determination of DOX. Their results showed that the modified electrodes had a high sensitivity of 185 *μ*A·mM^−1^ and a low detection limit of 0.02 *μ*M with a wide linear range of 0.02 *μ*M and −25 mM [26]. Yaghoobi et al. used a hybrid molecular nanostructure of glutamine and doxorubicin to identify human ovarian cancer cells. This system showed high sensitivity in detecting cancer cells [27]. A novel electrochemical platform for quantifying DOX in cancer cell lysates and plasma samples based on silver nanoparticles and a chitosan-coated glassy carbon electrode was proposed by Ehsani et al. This probe successfully monitored DOX concentrations in human biofluids and B16F10 cell lysates with high sensitivity [28]. Deepa et al. described an electrochemical method for determining DOX using cyclic voltammetry (CV) and differential pulse voltammetry (DPV). The sensor studied successfully monitored quality control, clinical analysis, and other therapeutic drugs [29]. Mi et al. reported a new sensor made of ZnS quantum dots in the shell, Ag nanoparticles (NPs), and CuInSe2 in the core with satisfactory results for DOX determination [30, 31]. In this research, we designed and constructed a novel biosensor with high functionality to detect DOX concentration in a targeted system. The performance of the biosensor in a biological environment will be investigated in detail.

## 2. Materials and Methods

### 2.1. Materials

In this study, all the required materials for the synthesis of advanced photocatalysts, including sodium oxalate (Na_2_C_2_O_4_), sodium nitrate (NaNO_3_), tungsten trioxide (WO_3_), ethanol (C_2_H_5_OH), potassium permanganate (KMnO_4_), hydrogen peroxide (H_2_O_2_), iron (III) chloride hexahydrate (FeCl_3_.6H_2_O), iron (II) sulfate heptahydrate (FeSO_4_.7H_2_O), ammonia (NH_3_), tartaric acid (C_4_H_6_O_6_), and glutamic acid (C_5_H_9_NO_4_), were supplied by Merck & Co.

### 2.2. Synthesis of Sodium Polytungstate

Sodium polytungstate (SPT) was synthesized through a multistage manufacturing process. In this regard, 0.9896 g sodium oxalate (Na_2_C_2_O_4_), 0.4954 g sodium nitrate (NaNO_3_), and 2.96 g tungsten trioxide (WO_3_) were first added to 150 mL of ethanol and stirred for three hours at 65°C under reflux, according to the following reaction:

(1) $$\begin{array}{c} {\text{Na}}_{2}{\text{C}}_{2}{\text{O}}_{4}+{\text{NaNO}}_{3}+\frac{3}{2}{\text{WO}}_{3}=\frac{3}{2}{\text{NaWO}}_{4}+2{\text{CO}}_{2}+\frac{1}{2}{\text{N}}_{2}{\text{O}}_{3} \end{array}$$

In the next step, the temperature of the resulting suspension was lowered to below 5°C in an ice bath. Then, 5 g of KMnO_4_ was slowly added to the mixture and stirred at room temperature for 30 minutes (500 rpm).

The temperature of the suspension was then raised to 25°C, and 10 mL of H_2_O_2_ was added dropwise and very slowly to the mixture. Stirring was then carried out at 500 rpm for 30 minutes. The resulting suspension was then placed in a tube furnace and annealed at 450°C for six hours under the flow of Ar. Then the resulting powder was ground and washed with ethanol and again placed in the tube furnace at 600°C for six hours for further recrystallization, followed by washing with ethanol after treatment. The resulting powder was then placed in the oven at 650°C for 6 h. Finally, the resulting powder was washed with ethanol, dried at 100°C for two hours, and ground to obtain a fine white powder of SPT.

### 2.3. Modification of Sodium Polytungstate through Green Protocol

A green protocol modified the purified SPT powder to improve its photocatalysis activity and organic components. First, 4.75 g FeCl_3_.6H_2_O and 3.89 g FeSO_4_.7H_2_O were added to a vessel containing deionized water and kombucha solvent in the ratio of 320 mL: 32 mL and stirred (500 rpm) for one hour at 80°C under reflux to obtain a homogeneous suspension. To prepare the kombucha solvent, a complete kombucha SCOBY (the mother kombucha SCOBY produces a complete kombucha SCOBY after 22 days) was washed well with DI and placed in a clean vessel in a dark and humid room. Then 500 ml of DI was added, and the kombucha SCOBY was fed with sugar every five days until the pH of the suspension dropped to about 1.5. The resulting suspension was used as green feed for the modification of SPT. In the next step, 1 g of well-purified SPT powder, 1 g of tartaric acid (C_4_H_6_O_6_), and 1 g of glutamic acid (C_5_H_9_NO_4_) were added to the resulting suspension and then stirred for 30 minutes under the same conditions. Then 40 mL of ammonia (NH_3_) was added dropwise to the resulting suspension and stirred for 24 hours at 80°C under reflux. Finally, the resulting powder was filtered with 0.22 *μ*m filler paper and washed several times with DI until the treated PST was neutralized with tartaric acid and glutamic acid (SPT-T-G) at a pH of 7.

### 2.4. Characterization

All prepared samples were characterized using several instruments, including Fourier transform infrared (FTIR) spectroscopy (Tensor II, Bruker, Germany) in the frequency range of 4000-400 cm^−1^, X-ray diffraction (XRD, Series S Max Finder Mira III, Tescan), and field-emission scanning electron microscopy (FESEM) with energy dispersive X-ray (EDX, Mira III, TESCAN).

### 2.5. Human Blood Sample Preparations

A phosphate buffer solution (PBS, 0.01 M) was obtained by mixing the corresponding K_2_HPO_4_ and KH_2_PO_4_. Doxorubicin was prepared in different concentrations. For this purpose, an exact amount of doxorubicin stock solution (0.2 M) was dissolved in a specific volume of buffer solution (pH = 7.4) and then stored at 4 °C in a dark place. Real samples of human blood plasma were provided by the Blood Transfusion Center (Iran) and frozen at −20°C. Then, 1 mL of H_2_SO_4_ (2 M) was added to the 2.0 mL of sample and centrifuged at 5000 rpm for 10 minutes to separate the plasma protein residues.

### 2.6. Apparatus

The electrochemical tests were carried out with a galvanostat/potentiostat AUTO LAB system (model PGSTAT302N, Netherlands) at room temperature. It was equipped with a standard cell and three electrodes, with a GC electrode (Metrohm) as a working electrode, a platinum wire (Metrohm) as a counter electrode, and Ag/AgCl (KCl 3M) (Metrohm) as a reference electrode. The data analysis was carried out with Nova 1.9 software.

## 3. Results and Discussions

### 3.1. Characterization

#### 3.1.1. FTIR Analysis

**Figure 1:**
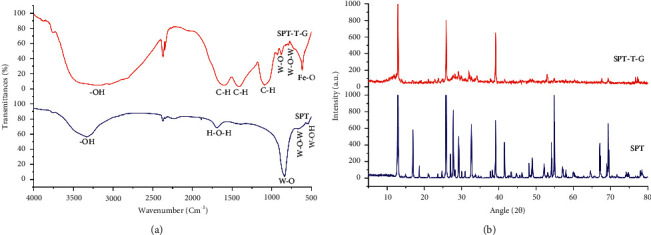
(a) FTIR and (b) XRD results of SPT and SPT-T-G specimens.

Figure 1(a) shows the FTIR spectrum of PTS and PTS/T/G as a function of the bonds formed. It was observed that the peaks in the range of 420 to 910 cm^−1^ correspond to the vibrations of the tungstate framework. The peaks that appeared in the FTIR spectrum of SPT can be assigned to the vibration of the W-OH group (522.14 cm^−1^), the vibration of W-O-W (649.6 cm^−1^), the vibration of W-O (886.67 cm^−1^), the vibration of the H-O-H group (1687.30 cm^−1^), and the hydroxyl functional groups (-OH) (3338.10 cm^−1^) [19]. In addition, Figure 1(a) (red color) shows the FTIR spectrum of the SPT-T-G sample. As can be seen in the figure, the peaks in the range between 530 and 630 cm^−1^ are associated with Fe-O vibrational stretching, confirming the presence of Fe in the structure of the composition, which covalently interacted with the SPT [32]. A sharp and intense peak appeared at 617.72 cm^−1^, representing the formed Fe-O functional groups. Other peaks appeared at 798.91 and 880.10 cm^−1^, indicating the W-O-W and W-O vibrations, respectively [19]. These peaks and their shifts demonstrate the successful synthesis of the biosensor, whose interaction and modification proceeded as expected. According to the FTIR spectrum analysis, other peaks have appeared, which are related to the sp2 alkene C-H band (disubstituted E) (928.29 cm^−1^), the in-plane stretching vibration of C-H (1083.28 cm^−1^), sp3 C-H bending (1411.70 cm^−1^), FeOO- (1599.42 cm^−1^), and the hydroxyl functional groups (-OH) (3186.64 cm^−1^) [21]. A sharp and broad peak of the functional group -OH represents the interaction and combination of different types of hydroxyl functional groups. Surprisingly, the SPT-T-G was not magnetic, which can be attributed to the role of kombucha solvent as it could change the nature of the synthesized Fe-based nanoparticles. To improve the biocompatibility of iron nanoparticles, the iron nanoparticles (III) should be converted into non-magnetic iron nanoparticles (II). The kombucha solvent can play this role because it contains a large amount of vitamin C and citric acid (CA) in its solvent. The citric CA acts as a capping agent and provides control of the particle size to be obtained. In situ covering of the surface of particles with CA reduces magnetic interparticle interactions, favoring their colloidal stability and making them attractive for biomedical applications. The increase in CA leads to a reduction in particle size and a decrease in interparticle interactions.

#### 3.1.2. XRD Analysis

The results of the XRD analysis are shown in Figure 1(b) and Table 1. SPT was successfully synthesized with several main chemical compounds, including C_12_O_8_, W_48_Na_136_O_328_, W_8_Na_8_O_28_, and Na_8_O_4_, with monoclinic cubic, orthorhombic, and cubic crystal structures, respectively. As shown in Table 1, the SPT POM consisted mainly of the cubic structure, except C_12_O_8_, which could have been formed by annealing the chemical compounds of the SPT with ethanol as a solvent. Together with the FTIR results, these data justify the successful synthesis of SPT POM. Similar to the FTIR analysis, the XRD results of SPT modified by the green protocol (i.e., SPT-T-G) also showed the successful interaction of SPT with the introduced organic chemical compounds, which may improve the sensitivity and interaction between the developed biosensor and the molecules. In this case, SPT-T-G contained various chemical compounds, including S_16_C_6_, W_48_Na_136_O_328_, W_8_Na_8_O_28_, Fe_16_, and N_1_C_l1_ with anorthic, cubic, orthorhombic, orthorhombic, and cubic crystal structures, respectively. The presence of the leading chemical compounds of SPT (i.e., W_48_Na_136_O_328_ and W_8_Na_8_O_28_) together with the introduction of nanoparticles of reduced iron (II) and materials of organic modifiers (i.e., S_16_C_6_ and N_1_C_l1_) in the structure of the synthesized SPT indicates that the SPT POM successfully interacted with and enhanced the selected materials. This resulted in a cubic SPT nanostructure with improved organic components, essential for the efficient absorption of pollutants from aqueous media. The noises may be referred to the nature of the arrangement of the layers mean SPT-T-G characterized by several noses by XRD. The noise will be reduced when the number of layers increases; thus, the noise can be explained. The amorphous has no sharp peaks, but the crystalline has sharp peaks and may be single or polycrystalline. Amorphous materials generally contain background noise, whereas crystalline materials contain peaks.

#### 3.1.3. FESEM Analysis

**Figure 2:**
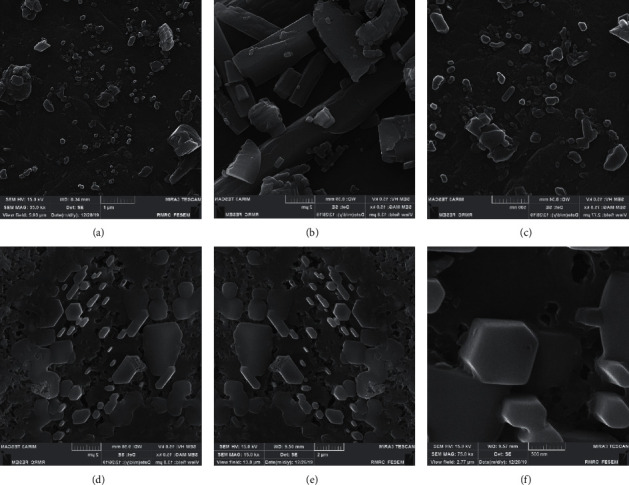
FESEM images of (a–c) SPT and (d–f) SPT-T-G at diverse scale bars. Red arrows show amorph particles, and blue ones show well-resolved cubic nanostructures.

Figure 2 shows the field-emission scanning electron microscopy (FESEM) images of SPT and SPT-T-G. As can be seen, SPT has a random morphology with different particle sizes. It can be seen that the modification of SPT with kombucha solvent, tartaric acid, and glutamic acid improved the morphology of SPT [33]. They also improved the structural characteristics of the final composition. SPT-T-G cubic has been characterized by FESEM techniques with energy dispersive X-ray (EDX). According to the FESEM images in Figure 2, the synthesized SPT-T-G particles have a cubic shape with an average particle size of about 51 nm, and their size distribution is wide. The observation of XRD and FESEM amorphous powder patterns may indicate the presence of amorphous, disordered crystalline material in the sample.

**Figure 3:**
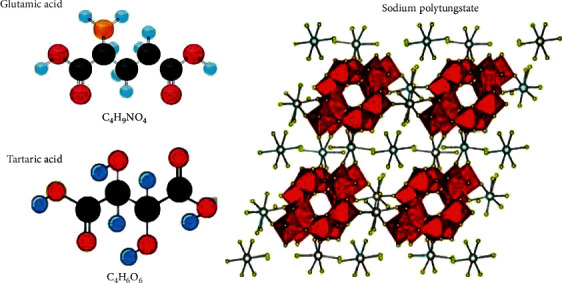
Chemical structure of glutamic acid, tartaric acid, and synthesized sodium polytungstate.

As shown in Figure 3, SPT-T-G had a well-defined cubic structure and some particles with random morphology throughout the sample, which was consistent with the data from XRD analysis. These data suggest that the modification of SPT by the green protocol significantly improved the morphology of the developed samples. The chemical structure of tartaric acid, glutamic acid, and the proposed crystalline structure corresponding to SPT is shown in Figure 3. The XRD analysis agreed well with the experimental data, and it was also found that the chemical structure of the compounds derived from SPT is cubic and that they were chemically improved. Further details can be found in Table 1.

#### 3.1.4. EDAX Analysis

**Figure 4:**
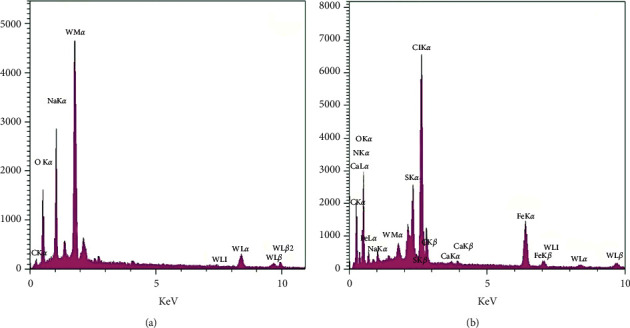
EDAX analysis of (a) SPT and (b) SPT-T-G.

The results of the EDAX analysis are shown in Figure 4 and Table 2. As can be seen in Figure 4 and Table 2, SPT consisted mainly of C, O, Na, and W, with W (%)/A (%) of 10.74/24.20, 31.94/54.01, 12.97/15.26, and 44.35/6.35, respectively, with O, Na, and W being the main constituents of SPT. The results of FTIR, XRD, and SEM analyses confirmed the successful synthesis of SPT with high purity. The summarised results showed that SPT-T-G contained more elements chemically compared to SPT due to the existence of more organic components in SPT formed during green synthesis and the conversion of iron nanoparticles (III) to iron nanoparticles (II) when kombucha was used as a solvent. In this case, SPT-T-G consisted of C, N, O, Na, S, Cl, Ca, Fe, and W, with Cl, S, and Fe having the highest intensities and weight fractions. These data are in good agreement with previously reported analyses (i.e., FTIR, XRD, and SEM) and confirm the interaction of SPT with the added organic modifiers.

### 3.2. Electrochemical Characterization of the Modified GCE

**Figure 5:**
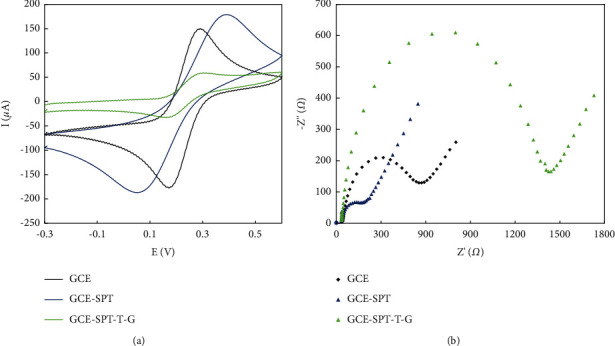
(a) CV curves and (b) Nyquist plots of bare GCE and modified GCE with STP and STP-T-P.

Given the CV response of the GCE to the electroactive probe [Fe(CN)6]3-/4- (5 mM), the electrode was significantly modified (Figure 5(a)). The bare GCE exhibited a well-defined reversible electrochemical response, while the electrode modified with POM1 exhibited a high peak separation (ΔEp) of 310 mV at 100 mVs^−1^. In this case, the POM1 materials showed slow electron transfer due to their structural effects. Furthermore, the oxidation and reduction potentials of the probe shifted to values that were more positive and negative, respectively. However, a decrease in the peak current of the probe was observed when the GCE surface was coated with POM2. They exhibited much slower electrode kinetics than POM1, where the electrode surface was completely blocked with increasing hydroxyl groups, inhibiting electron transfer between POM2 and Ferro/ferricyanide. This phenomenon could be due to the negative charge of the hydroxyl groups and the repulsion of the negatively charged [Fe(CN)6]3-/4-ions, which prevent the electrons from reaching the electrode surface. Considering a solution containing [Fe(CN)6]3-/4-ions with a frequency range of 105 to 10-2 Hz, a direct potential of 0.22 V, and an amplitude of ten mV at pH 7.4, impedance spectroscopy was performed and fundamentally evaluated to assess the ability of the surface to transfer electrons to different modified electrodes in this solution. The Nyquist plots from the EIS study and the electrical equivalent circuit obtained at the GCE electrode after each modification are shown in Figure 5(b). The R_ct_ value can be directly determined from the diameter of the semicircle of the Nyquist plots. It should be noted that the electron transfer resistance of the GCE, POM1-GCE, and POM2-GCE modified electrodes are close to 554.68 Ω, 212.65 Ω, and 1424.11 Ω, respectively. The modified electrodes exhibited a lower charge transfer resistance (Rct) than the bare electrode, according to the results achieved. These results mean that the electron transfer rate has increased due to the GCE coating and POM1 used. With POM2, on the other hand, the electron transfer capability decreased due to the negative charge of the hydroxyl groups and the repulsion of the negatively charged [Fe (CN)6]3-/4-ions.

### 3.3. Electrocatalytic and Sensing Performance

**Figure 6:**
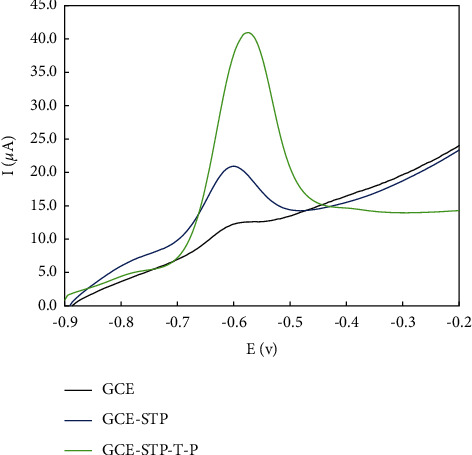
Electrochemical performance of bare and modified GCE with STP and STP-T-P via SWV technique.

**Figure 7:**
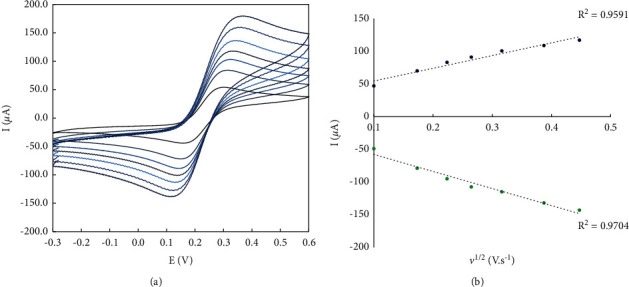
(a) Evaluation of the electrokinetic behavior of the developed sensor via CV method under variable scan rates for anodic and cathodic peak currents; (b) calibration curve of anodic and cathodic peak currents at diverse scan rates.

The electrocatalytic ability of the modified electrode was evaluated using the square wave voltammetry method with Vin ranging from −0.9 to −0.3 and 0.05M PBS. As shown in Figure 6, no significant peak current was observed at the unmodified GC electrode in the electrolyte containing 0.1 M doxorubicin solution. A current signal appeared at the electrode modified with POM and POM2 due to the doxorubicin solution. These results indicate that the POM2 deposited on the GC electrode made the electrode electroactive to detect doxorubicin. Upon further analysis, it was found that many peaks appeared on the modified electrode in the electrolyte without doxorubicin, indicating that the functional groups formed on the POM2-based sensor may enhance the electrocatalytic properties of the unmodified GC electrode. The kinetics of the electrochemical pathways can be assessed by cycling through different sampling rates of the voltammograms, as shown in Figure 7.  Figure 7(a) shows a regular increase in peak currents with increasing scan rate increases, suggesting that the process is controlled by diffusion [34].

### 3.4. Calibration Curve

**Figure 8:**
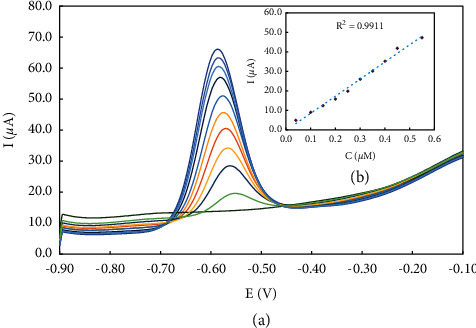
(a) SWV response of the modified GCE at a diverse concentration of DOX; (b) the inset plot shows the calibration curve of the process.

Optimal conditions were selected to detect different concentrations of doxorubicin. The current signal increased as the doxorubicin concentrations were gradually increased, as shown in Figure 8(a). In this context, a standard calibration curve was constructed by plotting the amount of doxorubicin against the magnitude of the signal current d (Figure 8(b)). The inset plot shows a linear calibration curve (I (*μ*A) = 87.862 C (*μ*M)−0.2865) between 0.04 and 0.55 *μ*M. As shown in Figure 8, the POM2-based sensor showed high sensitivity (evaluated by calculating the slope of the standard curve/area of GC [35] of 175.72 *μ*A·*μ*M^−1^). The limit of detection and limit of quantification were determined using the standard curve over 3 S/m and 10 S/m, respectively [36], and were 8.12 nM and 0.056 *μ*M, respectively. The accuracy of the approach used was evaluated and analyzed concerning doxorubicin adsorption on the POM2-modified electrode. Five measurements at three concentration levels (0.1 *μ*M, 0.3 *μ*M, and 0.5 *μ*M) were considered in this case. The relative standard deviation was in the range of 3.15%–5.12%, confirming the high repeatability of the sensor response for doxorubicin determination. Another GC electrode with the same surface area was used to test the robustness of the proposed approach. The relative standard deviation was 4.12% for the determination of 0.30 *μ*M doxorubicin, demonstrating the robustness of the POM2/GCE response.

A comparison of the results of the proposed method with other methods for quantifying DOX is shown in Table 3. Hashemi et al. recently developed a polyrhodanine biosensor functionalized with graphene oxide and iron oxide nanoparticles [37]. They integrated the kombucha solvent supernatant into the system to enhance biocompatibility and sensitivity. Their nanobiosensor revealed superior sensitivity and accuracy, where the sensitivity, lower limit of detection, and lower limit of quantification were measured to be 167.62 *μ*A·*μ*M^−1^·cm^−2^, 0.008 *μ*M and 0.056 *μ*M, respectively. This system detected DOX in the human blood plasma with high accuracy (>99%). As can be seen, the present work demonstrates a reasonable detection limit, and compared to some of the publications [38, 39], it shows a lower detection limit (8.12 nM) and quantification limit (56 nM). This fact validates the suitability of our sensor to detect DOX with high sensitivity.

### 3.5. Analytical Application

The applicability of the fabricated electrochemical sensor was evaluated in real spiked human plasma samples. Known DOX concentrations were added to the samples, and DPV measurements were performed using POM2/GCE. The results are presented in Table 4. The recovery values confirm the high accuracy of the DOX determination and the absence of matrix effects.

## 4. Conclusion

This study presented a novel biosensor with excellent functionality for detecting doxorubicin for targeted treatment. Using a green synthesis approach, we prepared a biosensor based on sodium polytangstate/polyoxometalate. The newly developed biosensor showed excellent physicochemical properties due to the unique and extraordinary components used in the biosensor structure. Our results indicate that the fabricated biosensor has a high potential for detecting DOX in the biological fluid. According to the results, the developed biosensor will be very useful in detecting DOX and can contribute to targeted treatment in a biological environment.

## Figures and Tables

**Table 1:** Data obtained from the XRD analysis for SPT and SPT-T-G nanostructures.

Sample	2ϴ (°)	d-spacing (Å)	Chemical compound	Plane (HKL)	Crystalline structure	Crystalline size (Å)	Micro strain (%)	Reference code
SPT	12.8645	6.8834	C_12_O_8_	(1 0 0)	Monoclinic	76.44221	4.503129	96-590-0040
	16.9443	5.28984	W_48_Na_136_O_328_	(1 1 1)	Cubic	115.4435	2.273826	96-591-0225
	21.0943	4.21383	W_8_Na_8_O_28_	(0 4 1)	Orthorhombic	77.27145	2.736177	96-900-7741
	25.7856	3.45558	C_12_O_8_	(0 1 –2)	Monoclinic	117.4237	1.490216	96-590-0040
	27.7323	3.22791	W_48_Na_136_O_328_	(0 2 2)	Cubic	117.5984	1.382735	96-591-0225
	32.8073	2.72904	W_48_Na_136_O_328_	(1 1 3)	Cubic	79.17859	1.755239	96-591-0225
	38.0566	2.37014	W_8_Na_8_O_28_	(1 9 0)	Orthorhombic	120.8277	0.980631	96-900-7741
	38.9109	2.33491	C_12_O_8_	(0 0 4)	Monoclinic	80.56572	1.457097	96-590-0040
	41.6281	2.1551	W_8_Na_8_O_28_	(1 1 2)	Orthorhombic	81.24937	1.352824	96-900-7741
	46.018	1.97334	Na_8_O_4_	(0 2 2)	Cubic	123.9971	0.784542	96-900-9064
	48.6876	1.87111	C_12_O_8_	(2 1 2)	Monoclinic	83.36645	1.136789	96-590-0040
	54.6524	1.68960	Na_8_O_4_	(1 1 3)	Cubic	85.4954	0.97741	96-900-9064
	64.8321	1.44351	W_48_Na_136_O_328_	(0 2 6)	Cubic	89.97712	0.78342	96-591-0225
	69.3549	1.35652	C_12_O_8_	(3 2 3)	Monoclinic	138.7834	0.485253	96-590-0040
	74.5461	1.27509	W_48_Na_136_O_328_	(1 1 7)	Cubic	114.6281	0.55437	96-591-0225

SPT-T-G	8.019	11.0591	S_16_C_6_	(0 0 1)	Anorthic	38.12231	14.49596	96-200-1129
	16.7378	5.3143	W_48_Na_136_O_328_	(1 1 1)	Cubic	76.80672	3.412017	96-591-0225
	20.3856	4.34683	W_8_Na_8_O_28_	(0 4 1)	Orthorhombic	77.09752	2.791682	96-900-7741
	23.0692	3.85634	Fe_16_	(0 0 4)	Orthorhombic	155.6814	1.197826	96-431-3217
	29.8097	2.97625	S_16_C_6_	(0 3 0)	Anorthic	157.7764	0.990606	96-200-1129
	32.752	2.79776	N_1_Cl_1_	(0 1 1)	Cubic	158.8874	0.898731	96-900-7495
	38.9324	2.32479	C_12_O_8_	(0 0 4)	Monoclinic	161.7505	0.785001	96-590-0040
	46.9255	1.94074	Fe_16_	(4 0 0)	Orthorhombic	166.6563	0.602292	96-431-3217
	58.3434	1.57447	N_1_Cl_1_	(1 1 2)	Cubic	174.8340	0.492332	96-900-7495

**Table 2:** Quantitative EDAX analysis of SPT and SPT-T-G.

Sample	Element	Intensity	W%	A%
SPT	C	38.6	11.04	24.50
	O	314.6	32.04	53.90
	Na	593.1	13.10	15.56
	W	234.0	44.95	6.73

SPT-T-G	C	145.6	29.09	39.05
	N	38.5	14.98	17.29
	O	305.9	32.09	34.11
	Na	33.4	0.54	0.41
	S	368.1	2.58	1.37
	Cl	1175.1	9.03	4.19
	Ca	17.21	0.16	0.06
	Fe	332.6	5.93	1.78
	W	43.1	5.46	0.50

**Table 3:** Comparison of different methods for determination of DOX.

Method	Limit of detection	Lower limit of quantitation	*R* ^2^	Ref.
DPV^a^	0.016 *μ*M	0.018 *μ*M	0.9971	[39]
DPV^a^	0.016 *μ*M	0.050 *μ*M	0.9971	[38]
EIS^B^	0.09 pg·mL^−1^	—	0.99	[40]
DPV	0.1 nM	—	0.9981	[41]
SWV	1.0 nM	—	0.9954	[42]
DPAdSV	2.8 nM	—	0.99	[43]
SWV	0.008 *μ*M	0.056 *μ*M	0.99	[37]
SWV	8.12 nM	56 nM	0.9911	This work

^a^Differential pulse voltammetry.^b^Electrochemical impedance spectroscopy.^C^Adsorptive stripping differential pulse voltammetry.

**Table 4:** Determination of DOX in human plasma samples (*n* = 6).

Samples	Added (*μ*M)	Found (*μ*M)	Recovery (RSD) (%)
Plasma	0.0	ND^*∗*^	—
	0.1	0.0925	92.50 ± 3.22
	0.3	0.2966	98.86 ± 2.95
	0.5	0.4721	94.42 ± 3.78

^*∗*^Not detectable.

## Data Availability

All data used to support the findings of this study are included within the article.

## References

[1] Mousavi S. M., Seyyed A. H., Sonia B. (2022). Hybrid of sodium polytungstate polyoxometalate supported by the green substrate for photocatalytic degradation of auramine-O dye. *Environmental Science and Pollution Research*.

[2] Zangeneh M. M., Norouzi H., Mahmoudi M., Goicoechea H. C., Jalalvand A. R. (2019). Fabrication of a novel impedimetric biosensor for label free detection of DNA damage induced by doxorubicin. *International Journal of Biological Macromolecules*.

[3] Gu J., Zhang L., Yuan X., Chen Y. G., Gao X., Li D. (2018). Synthesis and antibacterial activity of polyoxometalates with different structures. *Bioinorganic Chemistry and Applications*.

[4] López X., Carbo J. J., Bo C., Poblet J. M. (2012). Structure, properties and reactivity of polyoxometalates: a theoretical perspective. *Chemical Society Reviews*.

[5] Mousavi S. M., Hashemi S. A., Amani A. M., Saed H., Jahandideh S., Mojoudi F. (2017). Polyethylene terephthalate/acryl butadiene styrene copolymer incorporated with oak shell, potassium sorbate and egg shell nanoparticles for food packaging applications: control of bacteria growth, physical and mechanical properties. *Polymers from Renewable Resources*.

[6] Mousavi S. M., Hashemi S. A., Parvin N. (2021). Recent biotechnological approaches for treatment of novel COVID-19: from bench to clinical trial. *Drug Metabolism Reviews*.

[7] Mousavi S. M., Hashemi S. A., Kalashgrani M. Y. (2022). Bioactive graphene quantum dots based polymer composite for biomedical applications. *Polymers*.

[8] Čolović M. B., Lackovic M., Lalatovic J., Mougharbel A. S., Kortz U., Krstic D. Z. (2020). Polyoxometalates in biomedicine: update and overview. *Current Medicinal Chemistry*.

[9] Mitsui S., Ogata A., Yanagie H. (2006). Antitumor activity of polyoxomolybdate, [NH3Pri] 6 [Mo7O24]·3H2O, against, human gastric cancer model. *Biomedicine & Pharmacotherapy*.

[10] Thomadaki H., Karaliota A., Litos C., Scorilas A. (2007). Enhanced antileukemic activity of the novel complex 2,5-dihydroxybenzoate molybdenum (VI) against 2, 5-dihydroxybenzoate, polyoxometalate of Mo (VI), and tetraphenylphosphonium in the human HL-60 and K562 leukemic cell lines. *Journal of Medicinal Chemistry*.

[11] Boulmier A., Feng X., Oms O. (2017). Anticancer activity of polyoxometalate-bisphosphonate complexes: synthesis, characterization, in vitro and in vivo results. *Inorganic Chemistry*.

[12] Moucadel V., Laudet B., Barette C., Lafanechère L., Hasenknopf B. (2008). Identification of polyoxometalates as nanomolar noncompetitive inhibitors of protein kinase CK2. *Chemistry & Biology*.

[13] Müller C. E., Iqbal J., Baqi Y., Zimmermann H., Rollich A., Stephan H. (2006). Polyoxometalates—a new class of potent ecto-nucleoside triphosphate diphosphohydrolase (NTPDase) inhibitors. *Bioorganic & Medicinal Chemistry Letters*.

[14] Gholami A., Farjami F., Ghasemi Y. (2021). The development of an amperometric enzyme biosensor based on a polyaniline-multiwalled carbon nanocomposite for the detection of a chemotherapeutic agent in serum samples from patients. *Journal of Sensors*.

[15] Khosravi Ardakani H., Gerami M., Chashmpoosh M., Omidifar N., Gholami A. (2022). Recent progress in nanobiosensors for precise detection of blood glucose level. *Biochemistry Research International*.

[16] Gooding J. J. (2002). Electrochemical DNA hybridization biosensors. *Electroanalysis*.

[17] Gholami A., Hashemi S. A., Yousefi K. (2020). 3D nanostructures for tissue engineering, cancer therapy, and gene delivery. *Journal of Nanomaterials*.

[18] Mousavi S. M., Hashemi S. A., Bahrani S. (2021). Recent advancements in polythiophene-based materials and their biomedical, geno sensor and DNA detection. *International Journal of Molecular Sciences*.

[19] Bonanni A., Esplandiu M., Del Valle M. (2008). Signal amplification for impedimetric genosensing using gold-streptavidin nanoparticles. *Electrochimica Acta*.

[20] Mousavi S. M., Behbudi G., Hashemi S. A. (2021). Recent progress in electrochemical detection of human papillomavirus (HPV) via graphene-based nanosensors. *Journal of Sensors*.

[21] Chekin F., Myshin V., Ye R. (2019). Graphene-modified electrodes for sensing doxorubicin hydrochloride in human plasma. *Analytical and Bioanalytical Chemistry*.

[22] Mousavi S. M., Zarei M., Hashemi S. A. (2020). Asymmetric membranes: a potential scaffold for wound healing applications. *Symmetry*.

[23] Alarfaj N. A., El-Tohamy M. F. (2020). New functionalized polymeric sensor based NiO/MgO nanocomposite for potentiometric determination of doxorubicin hydrochloride in commercial injections and human plasma. *Polymers*.

[24] Gardikis K., Signorelli M., Ferrario C. (2017). Microbial biosensors to monitor the encapsulation effectiveness of doxorubicin in chimeric advanced drug delivery nano systems: a calorimetric approach. *International Journal of Pharmaceutics*.

[25] Mohkam M., Sara R.-A., Dariush S. (2016). Characterization and in vitro probiotic assessment of potential indigenous bacillus strains isolated from soil rhizosphere. *Minerva Biotecnologica*.

[26] Behravan M., Aghaie H., Giahi M., Maleknia L. (2021). Determination of doxorubicin by reduced graphene oxide/gold/polypyrrole modified glassy carbon electrode: a new preparation strategy. *Diamond and Related Materials*.

[27] Yaghoobi F., Davoudi Z. A. S., Karimi Shervedani R., Torabi M., Norouzi-Barough L. (2021). Highly sensitive mixed molecular nanostructures of glutamine and doxorubicin on gold for targeted recognition of A2780 cancer cells using impedance spectroscopy and quartz crystal microbalance. *Sensors and Actuators B: Chemical*.

[28] Ehsani M., Soleymani J., Mohammadalizadeh P. (2021). Low potential detection of doxorubicin using a sensitive electrochemical sensor based on glassy carbon electrode modified with silver nanoparticles-supported poly (chitosan): a new platform in pharmaceutical analysis. *Microchemical Journal*.

[29] Deepa S., Swamy B. K., Pai K. V. (2020). A surfactant SDS modified carbon paste electrode as an enhanced and effective electrochemical sensor for the determination of doxorubicin and dacarbazine its applications: a voltammetric study. *Journal of Electroanalytical Chemistry*.

[30] Mi G., Shi H., Yang M., Wang C., Hao H., Fan J. (2020). Efficient detection doxorubicin hydrochloride using CuInSe2@ ZnS quantum dots and Ag nanoparticles. *Spectrochimica Acta Part A: Molecular and Biomolecular Spectroscopy*.

[31] Mousavi S., Aghili A., Hashemi S., Goudarzian N., Bakhoda Z., Baseri S. (2016). Improved morphology and properties of nanocomposites, linear low density polyethylene, ethylene-co-vinyl acetate and nano clay particles by electron beam. *Polymers from Renewable Resources*.

[32] Kavanagh P., Leech D. (2006). Redox polymer and probe DNA tethered to gold electrodes for enzyme-amplified amperometric detection of DNA hybridization. *Analytical Chemistry*.

[33] Mousavi S. M., Hashemi S. A., Gholami A. (2021). Bioinorganic synthesis of polyrhodanine stabilized Fe3O4/graphene oxide in microbial supernatant media for anticancer and antibacterial applications. *Bioinorganic Chemistry and Applications*.

[34] Karimi Shervedani R., Bahrani S., SamieiForoushani M., Momenbeik F. (2017). Selective detection of dopamine in the presence of ascorbic and uric acids through its covalent immobilization on gold mercaptopropionic acid self assembled monolayer. *Electroanalysis*.

[35] Hashemi S. A., Mousavi S. M., Bahrani S., Ramakrishna S., Babapoor A., Chiang W. H. (2020). Coupled graphene oxide with hybrid metallic nanoparticles as potential electrochemical biosensors for precise detection of ascorbic acid within blood. *Analytica Chimica Acta*.

[36] Bahrani S., Razmi Z., Ghaedi M., Asfaram A., Javadian H. (2018). Ultrasound-accelerated synthesis of gold nanoparticles modified choline chloride functionalized graphene oxide as a novel sensitive bioelectrochemical sensor: optimized meloxicam detection using CCD-RSM design and application for human plasma sample. *Ultrasonics Sonochemistry*.

[37] Hashemi S. A., Mousavi S. M., Bahrani S. (2022). Bio-enhanced polyrhodanine/graphene oxide/Fe3O4 nanocomposite with kombucha solvent supernatant as ultra-sensitive biosensor for detection of doxorubicin hydrochloride in biological fluids. *Materials Chemistry and Physics*.

[38] Hashemzadeh N., Hasanzadeh M., Shadjou N., Eivazi-Ziaei J., Khoubnasabjafari M., Jouyban A. (2016). Graphene quantum dot modified glassy carbon electrode for the determination of doxorubicin hydrochloride in human plasma. *Journal of pharmaceutical analysis*.

[39] Hasanzadeh M., Hashemzadeh N., Shadjou N., Eivazi-Ziaei J., Khoubnasabjafari M., Jouyban A. (2016). Sensing of doxorubicin hydrochloride using graphene quantum dot modified glassy carbon electrode. *Journal of Molecular Liquids*.

[40] Rezaei B., Askarpour N., Ensafi A. A. (2014). A novel sensitive doxorubicin impedimetric immunosensor based on a specific monoclonal antibody–gold nanoaprticle–sol–gel modified electrode. *Talanta*.

[41] Guo Y., Chen Y., Zhao Q., Shuang S., Dong C. (2011). Electrochemical sensor for ultrasensitive determination of doxorubicin and methotrexate based on cyclodextrin graphene hybrid nanosheets. *Electroanalysis*.

[42] Peng A., Xu H., Luo C., Ding H. (2016). Application of a disposable doxorubicin sensor for direct determination of clinical drug concentration in patient blood. *International Journal of Electrochemical Science*.

[43] Jemelková Z., Zima J., Barek J. (2009). Voltammetric and amperometric determination of doxorubicin using carbon paste electrodes. *Collection of Czechoslovak Chemical Communications*.

